# On the sensitivity of centrality metrics

**DOI:** 10.1371/journal.pone.0299255

**Published:** 2024-05-09

**Authors:** Lucia Cavallaro, Pasquale De Meo, Giacomo Fiumara, Antonio Liotta

**Affiliations:** 1 Institute for Computing and Information Sciences, Radboud University, Nijmegen, The Netherlands; 2 DICAM Department, University of Messina, Messina, Italy; 3 MIFT Department, University of Messina, Messina, Italy; 4 Faculty of Engineering, Free University of Bozen-Bolzano, Bolzano, Italy; Università di Pisa, ITALY

## Abstract

Despite the huge importance that the centrality metrics have in understanding the topology of a network, too little is known about the effects that small alterations in the topology of the input graph induce in the norm of the vector that stores the node centralities. If so, then it could be possible to avoid re-calculating the vector of centrality metrics if some minimal changes occur in the network topology, which would allow for significant computational savings. Hence, after formalising the notion of centrality, three of the most basic metrics were herein considered (*i.e*., Degree, Eigenvector, and Katz centrality). To perform the simulations, two probabilistic failure models were used to describe alterations in network topology: *Uniform* (*i.e*., all nodes can be independently deleted from the network with a fixed probability) and *Best Connected* (*i.e*., the probability a node is removed depends on its degree). Our analysis suggests that, in the case of degree, small variations in the topology of the input graph determine small variations in Degree centrality, independently of the topological features of the input graph; conversely, both Eigenvector and Katz centralities can be extremely sensitive to changes in the topology of the input graph. In other words, if the input graph has some specific features, even small changes in the topology of the input graph can have catastrophic effects on the Eigenvector or Katz centrality.

## Introduction

The role of *node centrality metrics* has been extensively addressed over the years by researchers in a broad range of application domains. We recall, to cite a few among the most important ones, Computer Science, Sociology, Economics and Life Sciences with the goal to identify the most relevant elements in complex systems associated with both natural and artificial entities [1–3].

A complex system is usually modelled by means of a graph whose nodes correspond to the “*atomic*” components of the complex system itself (*e.g*., airports in an air transport system) and whose edges identify the interactions between these components (*e.g*., routes between airports). High-centrality nodes play a key role in determining the survival of a complex system: for example, if we assume that the graph is connected, then high-centrality nodes could correspond to points where many shortest paths converge. In this case, removing high-centrality nodes would cause many shortest paths to disappear, thus increasing the mutual distances between any pairs of nodes, while keeping the graph connected. In the worst case, the removal of high-centrality nodes could lead to the fragmentation of the graph into multiple disjoint components [2]. More formally, let *G* = 〈*N*, *E*〉 be the graph corresponding to a complex system. Herein, *N* is the set of nodes and *E* ⊆ *N* × *N* is the set of edges; we define a (node) *centrality metric* as a function $f_{\Theta }:N\to R^{+}$ that takes as input a node *i* ∈ *N* plus an (optional) set of parameters **Θ** and returns a non-negative real number as output. We stipulate that the larger *f*(*i*), the more central the node *i*.

Centrality measures are relevant to finding the best *k* *spreaders* in a social network: suppose, in fact, that each actor in a social network can send a message to his neighbours, who can recursively forward the message to their neighbours until (hopefully) the entire network is covered. Given an integer *k*, our aim is to identify the *top-k spreaders*; *i.e*., the set of *k* actors to choose as initial spreaders in order to reach the largest number of actors in the social network. Finding the top-*k* spreaders is a well-known NP-hard problem [4]; however, an approximate solution requires sorting the actors according to a certain centrality measure and selecting the *k* actors with the highest centrality. Previous studies show that nodes found by certain centrality measures are often able to approximate the optimal set of spreaders well [5].

Examples of centrality metrics, which are also the ones we considered in this paper, are: (i) the *Degree centrality*, which considers more influential the nodes having the higher number of neighbours, (ii) the *Eigenvector centrality*, in which nodes are important if connected with other relevant nodes, and (iii) the *Katz centrality*, which measure the relative influence of a node by computing the sum of the contributions associated with all walks (*i.e*., by contributions we mean a combined factor given by both the incident edge weights and an attenuation factor that depends to the distance from the original node and the others at a longer walking distance from it). The connectivity variation of *G* after the deletion of a fraction of nodes was investigated in the state-of-art [1, 6]. Such a problem is known as *site percolation problem*. Other works, such as the one proposed by Moore *et al*. [7] focused, instead, on the *bond percolation problem*, namely how graph robustness depends on the corruption of some of its edges. In order to compute the node centrality and to choose, hence, the most appropriate metric for the specific graph is important to have accurate knowledge of the *topology* of *G*. The easiest way to describe such topology is through the use of the *adjacency matrix* $\text{A}\in R^{\left|N|\times |N\right|}$ that is a square matrix that, if *unweighted* (*i.e*., if we consider only whether there is a connection between pair of nodes) and *undirected* (*i.e*., if there is an edge from *i* to *j* then we must admit the existence of an edge from *j* to *i*) graphs are considered, then the adjacency matrix will be binary and symmetric. Thus, **A**_*ij*_ = **A**_*ji*_ = 1 if and only if the pair of nodes *i* and *j* are connected by an edge, and **A**_*ij*_ = **A**_*ji*_ = 0 otherwise. If *weighted* graphs are under scrutiny, then **A**_*ij*_ equals the weight associated with the edge that connects the node *i* with the node *j*. If the graph is *directed*, then the existence of an edge from *i* to *j* does not necessarily imply the existence of an edge from *j* to *i*.

In this paper, we consider unweighted and undirected graphs and we work with *probabilistic failure models* [8] because failures of some components of the system associated with *G* and/or malicious external agents may cause an alteration of the topology of *G* through the deactivation of some nodes/edges. This alteration compromises the connectivity of *G* and, consequently, the functionality of the system that *G* represents that, in the worst circumstances, such connectivity loss may lead to stopping the functioning of the whole system. For instance, it is well known that the failure of some routers on the Internet could cause the interruption of communications on a global scale [3, 9, 10]. In addition, in the work of Callaway *et al*. [10] the authors asserted that nodes in a graph are *occupied* whether the physical elements to which they correspond are functioning and that the probability of occupation of each node can be uniform or it can depend on other parameters such as the node degree [10].

Despite the extensive literature supporting the study of graph robustness upon node (and/or edge) failures, there is not enough knowledge about the consequences that the deactivation of some nodes along with their incident edges would have on node centrality [3]. What is more, the problem is usually addressed by the first assumption that *all nodes* in *G* can fail. However, such an assumption is unrealistic; hence, a more general assumption would consist of dividing the set *N* of nodes into two subsets *N*′ and *N* − *N*′ such that only nodes in *N*′ could actually fail whereas nodes in *N* − *N*′ are preserved from failures as we proposed in our previous work [8].

We wish to fill this gap and, in particular, our goal is to check whether a particular centrality metric *f*_**Θ**_ is a *sensitive* function, that is, we are interested in evaluating how the centrality metrics behave if small perturbations occur in the graph. We define a *perturbation* as the action of making some nodes and edges of *G* inactive. More concretely, let us fix a threshold *τ* ∈ [0, 1] that represents the fraction of targeted nodes to be removed and suppose to randomly select a subset of nodes *N*′ ⊆ *N* of size |*N*|′ = ⌈*τ* × |*N*|⌉. We assume that only nodes in *N*′ can fail and, in addition, we stipulate that each node in *N*′ will be associated with a failure probability.

In this paper, we focus on two approaches to modelling node failure probability: the former, called *Uniform*, assumes that all nodes have a *constant* failure probability; the latter, called *Best Connected* assumes the probability a node survives a failure is proportional to its degree, that is, large degree nodes are more resistant to failures [8].

Note that we considered the *Best Connected* strategy because attempting to remove the most connected nodes has a higher impact in perturbing the network topology compared with, for instance, targeting the least connected ones.

In real-world scenarios the network perturbation could represent any type of network disturbances; for instance, in our previous work [8] we defined the two probabilistic failures above mentioned that might be used to simulate cascading failures (*e.g*., in power grids) [11], or the spreading confining of both viruses [12–14] and fake news [15, 16], or streamline the network, through pruning (*i.e*., by removing those nodes that have minor impact on the overall connectivity) [17].

Hence, we have that a perturbation transforms the adjacency matrix **A** of *G* into a new matrix $\tilde{\text{A}}=\text{A}-\Delta \text{A}$ where Δ**A** describes a perturbation of the original matrix **A**. As previously asserted, the matrix is symmetric and its entries are equal to 0 or 1. Indeed, for the sake of simplicity, we conducted our analyses on undirected and unweighted graphs.

We target to answering the following two research questions through not only by an experimental evaluation, which was conducted in our previous work [3] but also and above all throughout a detailed and focused mathematical investigation on the reasons behind: **RQ**_1_ *Under which conditions can we classify a perturbation as “small” in the Uniform and in the Best Connected probabilistic failure models, respectively?* **RQ**_2_ *How does the norm of the centrality vector vary in the two models?*

Thus, our research questions can be formulated as follows: if we fix an (arbitrarily small) threshold *ε* > 0, we wonder if there exists a threshold *δ* > 0 such that ‖*f*_**Θ**_(**A** − **ΔA**) − *f*_**Θ**_(**A**)‖_2_ < *ε* if we assume that ‖**ΔA**‖ is less than *δ*. Herein, ‖**ΔA**‖ is the *norm* of the matrix **ΔA** and ‖⋅‖_2_ is the euclidian norm of a vector [18]. In **RQ**_1_, we wish to understand under what circumstances the norm of a perturbation Δ**A** gets smaller than a threshold *ε* in the Uniform and in the Best Connected models. Because of the most commonly used matrix norms (*e.g*., the Frobenius and the spectral norm [18]) depend on the eigenvalues of the matrix itself, we have that the problem of estimating how **A** variations are related to the problem of estimating how the location of the eigenvalues of the matrix **A** change upon the failure of a subset of its entries. In turn, the problem of studying how the spectrum of **A** changes if some of its elements have been randomly perturbed has been extensively addressed in the literature [19–21].

This work extends our earlier study where we explored the effects of graph perturbations on centrality metrics [3]. In detail, while in our previous work, we just conducted an experimental analysis on the implications that graph perturbations have on nodes rankings according to fixed centrality metrics, herein we performed a in-depth theoretical study on the *sensitivity* of such metrics.

The paper is organised as follows. In ‘Materials and Methods’ Section we provide not only background materials on graphs and node centrality but also the literature review and the description of the datasets used to perform our analyses. In addition, there are also details on how to quantify the amount of perturbation and on whether the Degree, the Eigenvector, and the Katz centrality metrics can be considered as *continuous* function or not. Finally, this section provides details about the experimental setup.

Next, in the ‘Discussion’ Section we discuss the *sensitivity* of the three centrality metrics herein considered (*i.e*., Degree, Eigenvector, and Katz centrality) in our two proposed probabilistic failure models (*i.e*., Uniform and Best Connected), we illustrates the experiments performed to study how variation in ‖**ΔA**‖ impact on the three chosen centrality metrics by commenting the results obtained.

Lastly, in the ‘Conclusions and Future Works’ Sections the conclusions are drawn and the future research plans are illustrated.

## Materials and methods

This section provides details about the most relevant definitions of graphs and node centrality metrics. In addition, the relevant literature review is also described. Furthermore, there are two important subsections, namely ‘Quantifying the amount of perturbation in a graph’, and ‘Experimental Setup’. In the first one, we discuss under which conditions a perturbation ‖**ΔA**‖ can be classified as ‘small’. The second one described how our analyses were performed providing also details on the evaluation metrics used. Table 1 summarises all the symbols used in this section.

**Table 1:** Summary of all the symbols used to develop our experiments and theoretical background.

Symbol	Meaning
*G*	A Graph *G* = 〈*N*, *E*〉
$\tilde{\text{G}}$	Perturbed G
*G* ^⋆^	Erdős-Rényi (ER) random graph’s adjacency matrix
*N*	Set of nodes of *G*
*N*′	Set of nodes that could fail
*E*	Set of edges of *G*, with *E* ⊆ *N* × *N*
*A*	Adjacency Matrix
Δ*A*	Perturbation of A
$\tilde{\text{A}}$	Perturbed A
*Tr*(*A*)	Trace of **A**
*f*_**Θ**_	The function of a (node) centrality metric, $f_{\Theta }:N\to R^{+}$
**Θ**	Set of parameters
**d**	Vector for the Degree Centrality
**e**	The vector that stores the Eigenvector centrality rankings of nodes in G
**k**	Vector for the Katz Centrality
λ	Eigenvalue
λ_1_	Principal eigenvalue (*i.e*., the *spectral radius*)
*β*	Attenuation factor for the Katz Centrality
*τ*	Fraction of targeted nodes (in [0, 1])
*k*	Walk length
*Z*	Generic matrix with random variables
*p*	Probability for a node to fail, with *p* = [0, 1]
*S*_Δ*A*_	Set of perturbation matrix
*ε*	An (arbitrarily small) threshold with *ε* > 0
*δ*	Threshold, with *δ* > 0
*ψ*	Evaluation metric to quantify the amount of change in the adjacency matrix **A**
*ζ*	Evaluation metric to compute the deformation effect on the centrality metric under scrutiny
*z*	Parameter (in [0, 1]) that measures how much the size of *G*′ has been reduced with respect to *G*
*η*	probability (in [0, 1]) that two randomly chosen nodes are connected by an edge

### Basic definitions on graphs

We define a graph *G* (or network) as a pair *G* = 〈*N*, *E*〉 in which *N* is the *set of nodes* (or vertices) and *E* ⊆ *N* × *N* is the *set of edges* (or links). Herein, we conducted our experiments on undirected and unweighted graphs, which means that for each edge 〈*i*, *j*〉 ∈ *E*, we have 〈*j*, *i*〉 ∈ *E* (*i.e*., undirected graphs) and that the edges have all the same cost (*i.e*., unweighted graphs). We define the *order* of a graph as the number *n* = |*N*| of its nodes and the *size* of a graph as the number *m* = |*E*| of its edges.

We say that a graph is *sparse* (resp., *dense*) if *m* = *O*(*n*) (resp., *m* = *O*(*n*^2^)).

Given a node *i* ∈ *N*, we define the *neighbourhood* *N*(*i*) of *i* as the set of nodes linked to *i*, namely *N*(*i*) = {*j* ∈ *N*: 〈*i*, *j*〉∈*E*}.

A *walk* of length *k* (being *k* a non-negative integer) is an ordered sequence of nodes 〈*i*_0_, *i*_1_, …, *i*_*k*_〉 such that consecutive nodes in the sequence are tied by an edge. We use the term *path* for walks that do not have repeated nodes. A walk is *closed* if it starts and ends at the same node.

Each unweighted graph *G* of order *n* is associated with an *n* × *n* matrix **A** called *adjacency matrix* such that **A**_*ij*_ = 1 if and only if 〈*i*, *j*〉 ∈ *E*, 0 otherwise. If the graph is undirected, then its adjacency matrix is *symmetric*; hence, all its eigenvalues λ_1_ ≥ λ_2_ ≥ … ≥ λ_*n*_ are real. The largest eigenvalue λ_1_ of **A** is also called its *principal eigenvalue* or *spectral radius* of *G*. Moreover, the corresponding eigenvectors **e**_1_, …, **e**_*n*_ will form an orthonormal basis in $R^{n}$ [22]. Eigenpairs 〈λ_*i*_, **e**_*i*_〉 are formed by the eigenvalue λ_*i*_ and its associated eigenvector **e**_*i*_.

The adjacency matrix is relevant to describe many graph properties: for instance, the matrix **A**^2^ where ${\text{A}}_{ij}^{2}=\sum_{r=1}^{n}{\text{A}}_{ir}{\text{A}}_{rj}$, gives the number of walks of length two going from *i* to *j*. By induction, for any positive integer *k*, the matrix **A**^*k*^ will give the number of closed (resp., distinct) walks of length *k* between any two nodes *i* and *j* if *i* = *j* (resp., if *i* ≠ *j*) [23].

### Node centrality

We define the *centrality of a node* as a function $f_{\Theta }:N\to R^{+}$ which takes as input a node *i* ∈ *N* along with an (optional) set of parameters **Θ** and it returns a non-negative real number as output.

The centrality *f*_**Θ**_(*i*) of a node *i* assesses the “importance” of *i* within *G*. Since there are many different ways to can interpret the notion of importance, there are consequently many definitions of centrality metrics. In the following sections, we explore the most used ones that are the also metrics we considered for our experiments.

#### The Degree centrality

The oldest and simplest definition of centrality is the *Degree centrality*. The *degree* *d*_*i*_ of *i* is equal to the number of neighbours of *i*, namely *d*_*i*_ = |*N*(*i*)|. Its centrality metric privileges nodes which are well connected with all other nodes in the graph and it is a *local centrality* measure in the sense its computation does not require to know the whole graph topology. In the following, we use the vector $\text{d}\in R^{N}$ to store the Degree centrality of the nodes in *G*. If we denote as $1\in R^{n}$ the vectors with all entries equal to one, then the Degree centrality **d** can be computed as follows:

$$\begin{array}{c} \text{d}=\text{A}1 \end{array}$$ (1)

#### The Eigenvector centrality

A further, interesting centrality metric is the *Eigenvector centrality* [1]. Unlike the Degree, the Eigenvector centrality of a node *i* does not depend on the number of neighbours of *i* but *on the importance of these neighbours*. Specifically, the Eigenvector centrality can be recursively computed through the following equation:

$$\begin{array}{c} \text{Ae}=\lambda \text{e} \end{array}$$ (2)

Where $\text{e}\in R^{n}$ is the vector storing the Eigenvector centrality rankings of nodes in *G*. Eq 2 does not admit a unique solution and, in particular, any eigenpair 〈λ_*l*_, **e**_*l*_〉 satisfies Eq 2. However, if we assume that the graph *G* is undirected and connected, we can take the largest eigenvalue λ_1_ and the corresponding eigenvector **e**_1_ (also known as *principal eigenvector*); by the Perron-Frobenius theorem [24] we have that all the components of **e**_1_ are positive and, thus, we can interpret the *i*-th component of **e**_1_ as the Eigenvector centrality of **A** itself.

#### The Katz centrality

A third important measure to consider is the so-called *Katz coefficient* [1]. The Katz centrality of a node *i* counts all walks beginning at *i*; each walk of length *k* is associated with a weight equal to *β*^*k*^, being the parameter *β* called the *attenuation factor* [25]. We can introduce a vector $\text{k}\in R^{n}$ which stores the Katz centrality of the node *i* in its *i*-th component; the vector **k** is defined as follows:

$$\begin{array}{c} \text{k}=(\text{I}+\beta \text{A}+\beta^{2}{\text{A}}^{2}+\ldots )1=(\sum_{k=0}^{+\infty }\beta^{k}{\text{A}}^{k})1 \end{array}$$

being $\text{I}\in R^{n\times n}$ the identity matrix. If we assume that $\beta <\frac{1}{\lambda_{1}}$ then the series $\sum_{k=0}^{+\infty }\beta^{k}{\text{A}}^{k}$ (often called *Neuman series*) is convergent and its sum equals to the inverse of the matrix **I** − *β* **A** [25]:

$$\begin{array}{c} \text{k}=(\sum_{k=0}^{+\infty }\beta^{k}{\text{A}}^{k})1=(\text{I}-\beta \text{A}{)}^{-1}1 \end{array}$$

Because of the equation above, we can interpret **k** as the solution of the following system of linear equations:

$$\begin{array}{c} (\text{I}-\beta \text{A})\text{k}=1 \end{array}$$

The matrix **V** = **I** − *β* **A** is *symmetric*, since it is obtained as the difference of two symmetric matrices. In addition, the constraints imposed on *β* imply that **V** is *positive definite* (*i.e*., all its eigenvalues are positive), and, thus, *non-singular*; indeed, if λ_*i*_ is an eigenvalue of **A** associated with the eigenvector **e**_*i*_ (that is **Ae**_*i*_ = λ_*i*_**e**_*i*_), we have that **e**_*i*_ is also an eigenvector of **V** corresponding to the eigenvalue 1 − *β*λ_*i*_:

$$\text{V}{\text{e}}_{i}=(\text{I}-\beta \text{A}){\text{e}}_{i}=\text{I}{\text{e}}_{i}-\beta \text{A}{\text{e}}_{i}={\text{e}}_{i}-\beta \lambda_{i}{\text{e}}_{i}=(1-\beta \lambda_{i}){\text{e}}_{i}$$

Since $\beta <\frac{1}{\lambda_{1}}$ and λ_*i*_ ≤ λ_1_ for all *i* = 1, …, *n*, we have that

$$\beta \lambda_{i}<\frac{\lambda_{i}}{\lambda_{1}}\to 1-\beta \lambda_{i}>1-\frac{\lambda_{i}}{\lambda_{1}}>0$$

In other words, all eigenvalues of **V** are strictly positive, provided that *β* is strictly less than $\frac{1}{\lambda_{1}}$ and this is enough to state that **V** is non-singular. In the following, we will use the interpretation of the Katz coefficient as the solution of a system of linear equations to accurately estimate the deviation of the Katz coefficient when some nodes (and the corresponding edges) are removed from a graph.

### Datasets

In this paper, we considered four real networks namely: (i) *Twitch-PT* [26], a social network of Twitch users collected in Spring 2018. Twitch is a video live streaming service that provides services such as video game live streaming as well as broadcasts of e-sports competitions. Nodes are Twitch users located in Portugal and edges are mutual follower relationships between them. (ii) *Twitch-UK* [26]: this dataset has the same structure and meaning of *Twitch-PT* but its nodes correspond to Twitch users from Portugal. (iii) *AstroPH* [27], a graph recording scientific collaborations between authors who submitted papers to the Astro-Physics category in the e-print arXiv service. Herein, nodes are associated with authors and there is an edge from nodes *i* and *j* if and only if authors *i* and *j* wrote a paper together. (iv) *Cond-Mat* [27], a collaboration network depicting scientific collaborations between authors who submitted papers to the Condense Matter category in ArXiv. Nodes and edges have the same meaning in *AstroPH*.

We have chosen two different social networks from the same platform (*i.e*., Twitch) because they came from two different cultural backgrounds (*i.e*., Portuguese and English) and, thus, the top-ranked nodes may be different as the network topology itself because the interests could vary in the two countries. Furthermore, we have not used a wider range of datasets because, as shown later in the ‘Discussion’ Section, the dataset chosen does not significantly affect our simulation as the parameters herein investigated are not highly dependent on the network topology used.

Table 2 shows more detailed network sizes in terms of number of nodes and edges.

**Table 2:** Characteristics of the dataset used. The table contains the name of the datasets with their corresponding references jointly with the total number of nodes and edges per each dataset.

Dataset	Nodes	Edges
Twitch-PT [26]	1 912	31 299
Twitch-EN [26]	7 126	35 324
AstroPh [27]	18 771	198 050
Cond-Matt [27]	30 460	120 029

### Related works

One of the early approaches devoted to analyzing how alterations in graph topology affect the ranking generated by a centrality metric is due to Costenbader and Valente [28]. The authors took random samples from an input directed graph and they varied the proportion of sampled nodes; specifically, they started by sampling 80% of the available nodes and gradually decreasing the sampling proportion by 10%. The sampling process stopped if the sampled network contained less than 10% of the input nodes. At each sampling level, Costenbader and Valente [28] computed how the centrality in the original graph and in the sampled graph were correlated. The authors found that some centrality metrics such as the in-degree and the Eigenvector centrality in the original and sampled graph were highly correlated; for other centrality metrics such as the out-degree), they observed a quicker decline in average correlation as a function of the sampling rate.

A nice extension of the work done by Costenbader and Valente is due to Borgatti *et al*. [29], who studied whether some centrality metrics can be regarded as robust if random errors occurred in the graph topology. The authors generated random graphs of different sizes and densities and they considered four types of errors, namely, edge deletion, node deletion, edge addition, and node addition. The main results of the study proposed in [29] show that the accuracy of centrality measures declines in a predictable function as a function of the amount of error. The approaches above assume that graph topology is fully specified and that some sampling task has been applied to it.

Another relevant approach that should be mentioned is the work of Kossinets [30], which investigated the impact of missing data on the structural properties of social networks. The author performed sensitivity analyses to discuss three principal missing data mechanisms: network boundary specification (non-inclusion of actors or affiliations), survey non-response, and censoring by node degree (fixed choice design), examining their impact on a scientific collaboration network. One of the most relevant outcomes was that, under certain circumstances, the largest component in a network assortatively mixed by node degree is less robust to random deletion of nodes than in a comparable neutral network.

Frantz *et al*. [31], instead, focused their efforts on examining the role of network topology, in conjunction with the type and amount of error, to determine the robustness of centrality metrics under uncertainty. The authors’ findings suggest that making *a priori* classification of the topology of a the network provides important additional information about the probabilistic reliability of the network measures that are computed over the observed data.

A different perspective has been considered by Diesner *et al*. [32]; herein, the authors consider social networks constructed from records of social interactions. Potential ambiguities of social entities may greatly affect the network construction process: for instance, nodes associated with the same string could be wrongly merged despite they are associated with distinct individuals. Diesner *et al*. [32] investigated the robustness of some centrality metrics such as the in-degree and they found that some graph statistics are heavily influenced by incorrect data but the process of detecting the most important node was robust to disambiguation flaws. Such a result implies that highly central individuals will still continue occupying a prominent ranking if we heavily corrupt input data. In line with Diesner *et al*. [32], Mishra *et al*. [33] studied to what extent flawed author name disambiguation can lead to wrong conclusions about gender bias in science.

More recently, some authors extended the concept of adversarial attack, initially developed in the context of machine learning systems, to social networks. The goal is to define the smallest amount of modification to apply on the observed graph to modify the node ranking produced by a particular centrality metric. In detail, the authors performed some experiments and they found that there is a small set of moves that result in the adversary achieving their objective, and this set is smaller for decreasing centrality metrics than for increasing them.

### Quantifying the amount of perturbation in a graph

In this section, we discuss under which conditions a perturbation ‖**ΔA**‖ can be classified as small. Hence, we define a *perturbation* as the action of making some nodes and edges of *G* inactive.

To do so, we fixed a threshold *τ* ∈ [0, 1] that represents the fraction of targeted nodes to be removed and we supposed to randomly select a subset of nodes *N*′ ⊆ *N* of size |*N*|′ = ⌈*τ* × |*N*|⌉ and we assume that only nodes in *N*′ can fail. Finally, we assume that each node in *N*′ will be associated with a failure probability.

We have several options to compute the norm of a matrix; the two most used ones are the *spectral norm* and the *Frobenius norm* [18]

The spectral norm ‖**A**‖_2_ of a matrix **A** is the largest singular value of **A**, namely the square root of the largest eigenvalue of the matrix **A** * **A**, where A* is the conjugate transpose of **A**. If we restrict our attention to real matrices then A* coincides with the transpose **A**^*T*^ of **A**. In our case, the matrix **ΔA** is, by construction, square and symmetric and, thus, the spectral norm of ‖**ΔA**‖_2_ coincides with its largest eigenvalue λ_1_ [18].

The Frobenius norm of a matrix **A** is defined as follows:

$$\begin{array}{c} \|\text{A}{\|}_{F}=\sqrt{\sum_{i=1}^{n}\sum_{j=1}^{n}{\text{A}}_{ij}^{2}} \end{array}$$

It is possible to show that the Frobenius norm of **A** equals the square root of the sum of the squares of its singular values *σ*_*ℓ*_(**A**), namely $\|\text{A}{\|}_{F}=\sqrt{\sum_{\ell =1}^{n}\sigma_{\ell }^{2}}$. Because of we manage only undirected graphs, then the matrix **A** is square and symmetric, and, thus, its singular values coincide with its eigenvalues and, thus, $\|\text{A}{\|}_{F}=\sqrt{\sum_{i=1}^{n}\lambda_{i}^{2}}$. Finally, because of the sum of the eigenvalues of a matrix **A** is equal to the *trace* *Tr*(*A*) of **A** (*i.e*., the sum of the elements on its main diagonal) we have that:

$$\begin{array}{c} \|\text{A}{\|}_{F}=\sqrt{Tr({\text{A}}^{2})} \end{array}$$

The Frobenius norm of **A** and its spectral norm are related as follows:

$$\|\text{A}{\|}_{F}=\sqrt{\sum_{i=1}^{n}\sum_{j=1}^{n}{\text{A}}_{ij}^{2}}=\sqrt{\sum_{\ell =1}^{n}\lambda_{\ell }^{2}}\geq \sqrt{\lambda_{1}^{2}}=\lambda_{1}=\|\text{A}{\|}_{2}$$

We finally recall that both the Frobenius norm and the spectral norm are *submultiplicative*, namely ‖**AB**‖ ≤ ‖**A**‖ ‖**B**‖ where **A** and **B** are two arbitrary matrices and **AB** is their product.

Previous research works focused on estimating the spectrum of a matrix with random entries [19, 34]. In this paper, we assume to work with *small perturbations*, and our assumption is reasonably equivalent to considering **ΔA** as a sparse matrix. We recall that efficient techniques are available to compute the largest eigenvalue λ_1_ of the **ΔA** (see Trefethen and Bau [35] for a detailed survey of available methods), which coincides with its spectral norm (because of **ΔA** is a symmetric matrix).

The calculation of the Frobenius norm requires *O*(*n*^2^) steps; recently, some researchers [36] have applied sampling techniques to approximate the Frobenius norm and they have shown that even a small number of samples is sufficient to obtain an accurate estimate.

### Experimental setup

Herein, we briefly explain the experimental setup to allow the replicability of our results. To develop the experiments we have used some scripts we implemented in Python by using the classic libraries for dealing with graphs (*e.g*., NetworkX, NumPy, Pandas, to cite some). Given a graph *G* = 〈*N*, *E*〉 we assume that each node *i* ∈ *N* is associated with a *removal probability* *p*_*i*_, or, equivalently, with a *survival probability* *q*_*i*_ = 1 − *p*_*i*_.

We consider two options for modelling the removal probability *p*_*i*_ namely: Uniform, in which *p*_*i*_ is a constant and Best Connected, in which *p*_*i*_ is proportional to the degree of *i* [8]. In what follows, let $\text{p}\in R^{\left|N\right|}$ be a vector such that the *i*-th component of **p** equals the failure probability *p*_*i*_.

In our model, we also define and introduce a parameter *τ* ∈ [0, 1], which controls the percentage of nodes which can fail (or, equivalently, 1 − *τ* specifies the fraction of nodes preserved from failures). Specifically, we assume to draw, uniformly at random, a subset *N*′ ⊆ *N* of nodes from *N* of size equal to ⌈*τ* × |*N*|⌉. We assume that only nodes in *N*′ can fail while nodes located in *N* − *N*′ are preserved from failures: in other words, if a node *i* ∈ *N*′, then *i* will fail with probability *p*_*i*_; in contrast, if *i* ∈ *N* − *N*′, then its failure probability will automatically set equal to 0. Our model extends the traditional site percolation model [1, 6, 7], which assumes that *τ* = 1; *i.e*., it assumes that all nodes can fail.

The overall node protocol failure can be described as a *two-stage process*: in the first stage, we build the set *N*′ of nodes that can fail; in the second stage, we select, with probability *p*_*i*_, a node *i* with *i* ∈ *N*′. Selected nodes are deleted from *G* along with their edges. The process above yields a graph $\tilde{G}$ with adjacency matrix $\tilde{\text{A}}$. We define as *perturbation* the matrix $\Delta \text{A}=\tilde{\text{A}}-\text{A}$.

Let *f*_**Θ**_ be a centrality metric that can depend on an optional set of parameters **Θ** (for instance, **Θ** could coincide with the attenuation parameter *β* in the Katz coefficient). We are concerned with estimating how an alteration on the topology of *G* affects the centrality scores produced by *f*_**Θ**_. In particular, we are interested in determining under which conditions a “small” perturbation ‖**ΔA**‖ would also cause a small variation in ‖*f*_**Θ**_‖. Herein, the symbol ‖⋅‖ is the *norm of a matrix* [18]. In Section Quantifying the amount of perturbation in a graph we provided some examples of norms of matrices.

We are now able to provide the following definition:

**Definition 1**. *Let G be a graph with n nodes and m edges and let* $\text{A}\in R^{n\times n}$ *be its adjacency matrix. Let us consider a perturbation associated with the matrix* **ΔA** *which produces the perturbed graph* $\tilde{G}$ *with adjacency matrix* $\tilde{\text{A}}=\text{A}-\Delta \text{A}$. *Let* $f_{\Theta }:N\to R^{+}$ *a centrality metric. We say that f*_**Θ**_ *is continuous if for every ε* > 0 *there exists δ* > 0 *such that*:

$$\begin{array}{c} \|f_{\Theta }(\tilde{\text{A}})-f_{\Theta }\left(\text{A}\right)\|<\varepsilon \;\text{if}\;\|\tilde{\text{A}}-\text{A}\|<\delta \end{array}$$

#### Evaluation metrics

In this paper we target at answering the following two research questions: **RQ**_1_ *under which conditions can we classify a perturbation as “small” in the Uniform and in the Best Connected models, respectively?* **RQ**_2_ *How does the norm of the centrality vector vary in the Uniform and in the Best Connected models?* Hence, we considered two metrics to evaluate the results obtained, namely: (i) *ψ* to quantify the graph perturbation (*i.e*., to quantify the amount of change in the adjacency matrix **A**) to address **RQ**_1_, (ii) and *ζ*, to address **RQ**_2_, to compute the deformation effect on the centrality metric under scrutiny. The first metric is computed as follows:

$$\begin{array}{c} \psi =\frac{\left\|\Delta \text{A}\right\|}{\left\|\text{A}\right\|} \end{array}$$ (3)

where **ΔA** is the perturbation matrix, and **A** is the adjacency matrix.

As previously said, it quantifies the amount of change in the adjacency matrix **A** due to the application of a perturbation. Thus, this metric was used to evaluate the graph perturbation.

In the Uniform model the matrix **ΔA**_*F*_ depends on both the parameters *p* and *τ*, while in the Best Connected model the matrix **ΔA**_*F*_ only depends on *τ*.

In both the Uniform and Best Connected models, we have that 0 ≤ *ψ* ≤ 1.

We also computed the deformation of centrality metrics. Specifically, let *f*_**Θ**_(**A**) (resp., $f_{\Theta }(\tilde{\text{A}}$)) be the vector containing the centrality scores computed via the function *f*_**Θ**_(⋅) on the input (resp., modified) adjacency matrix **A** (resp., $\tilde{\text{A}}$). We defined the following parameter:

$$\begin{array}{c} \zeta =\frac{\left\|f_{\Theta }\left(\tilde{\text{A}}\right)-f_{\Theta }\left(\text{A}\right)\right\|}{\left\|f_{\Theta }\left(\text{A}\right)\right\|} \end{array}$$ (4)

where *f*_**Θ**_ is the centrality of a node $f_{\Theta }:N\to R^{+}$ that takes as input a node *i* ∈ *N* plus an optional set of parameters **Θ** and, as previously asserted, returns a non-negative real number as output: the larger *f*(*i*), the more central the node *i*.

## Discussion

This section firstly provides a discussion about the *sensitivity* behaviour of the three centrality metrics under scrutiny (*i.e*., Degree, Eigenvector, and Katz centrality) in the two probabilistic failure models (*i.e*., Uniform and Best Connected). Next, the experiments we carried out to validate our model are reported. More specifically, this second part of the section is divided into three parts: (i) ‘When a perturbation is small’, (ii) ‘How the centrality metrics vary in the two probabilistic failure models’, and (iii) ‘Take-home message’. The first two are aimed to answer our two main research questions (*i.e*., **RQ**_1_ *Under which conditions can we classify a perturbation as “small” in the Uniform and in the Best Connected models, respectively?* **RQ**_2_ *How does the norm of the centrality vector vary in the Uniform and in the Best Connected models?*), whereas the third one summarises the most relevant outcomes.

### The *sensitivity* of the centrality metrics

In this section, we discuss the *sensitivity* of the three centrality metrics herein considered (*i.e*., Degree, Eigenvector, and Katz centrality) in our two proposed probabilistic failure models (*i.e*., Uniform and Best Connected).

#### The *sensitivity* of Degree centrality

We start by discussing the *sensitivity* of the Degree centrality in the Uniform and Best Connected models. Let **d** and $\tilde{\text{d}}$ be the array of Degree centrality rankings in *G* and $\tilde{G}$, respectively.

From Eq 1 we have:

$$\begin{array}{c} \begin{array}{c} \|\tilde{\text{d}}-\text{d}{\|}_{2}=\|\tilde{\text{A}}1-\text{A}1{\|}_{2}=\\ =\|(\tilde{\text{A}}-\text{A})1{\|}_{2}\leq \|\tilde{\text{A}}-\text{A}{\|}_{F}\|1{\|}_{2}=\\ =\|-\Delta \text{A}{\|}_{F}\|1{\|}_{2}=\sqrt{n}\|\Delta \text{A}{\|}_{F} \end{array} \end{array}$$

where the last inequality derives from the submultiplicativity of the Frobenius norm and from the fact that $\|1{\|}_{2}=\sqrt{n}$. Due to the monotonicity of expectation, we get

$$\begin{array}{c} \text{E}\;\left[\|\tilde{\text{d}}-\text{d}{\|}_{2}\right]\leq \sqrt{n}E\;\left[\|\Delta \text{A}{\|}_{F}\right] \end{array}$$

As a consequence, if we assume that ‖**ΔA**‖_*F*_ < *ε* we can pick $\delta =\frac{\varepsilon }{\sqrt{n}}$ to ensure $\text{E}\;[\|\text{d}-\tilde{\text{d}}{\|}_{2}]\leq \delta$, which proves the *sensitivity* of the Degree centrality.

#### The *sensitivity* of Eigenvector centrality

In this section, we present our results about the *sensitivity* of the Eigenvector centrality.

Our goal is to measure $\left|\right|\tilde{\text{e}}-\text{e}\left|\right|$, where $\tilde{\text{e}}$ (resp., **e**) is the leading eigenvector of the adjacency matrix of the perturbed graph $\tilde{G}$ (resp., the input graph *G*).

A useful tool for our purposes is the Davis-Kahan theorem [37–39], which we briefly explain below. In particular, let us focus on two arbitrary matrices $\Sigma ,\tilde{\Sigma }\in R^{p\times d}$ and let us construct the product $\Lambda ={\tilde{\Sigma }}^{T}\Sigma$, which has singular values *σ*_1_ ≥ *σ*_2_ ≥ … ≥ *σ*_*d*_. We define the vector $\text{w}\in R^{d}$ of *principal angles* **w** = [arccos(*σ*_1_), …, arccos(*σ*_*d*_)]. We also define the *d* × *d* diagonal matrix $\Theta (\Sigma ,\tilde{\Sigma })$ whose entries are equal to the entries of the vector **w** and the matrix $\text{sin}\left(\Theta \right)\left(\Sigma ,\tilde{\Sigma }\right)$ obtained by applying the sin function to all the entries of the matrix $\Theta (\Sigma ,\tilde{\Sigma })$. The distance between the column spaces spanned by matrices **Σ** and $\tilde{\Sigma }$ can be measured through the expression $||\text{sin}\left(\Theta \right)\left(\Sigma ,\tilde{\Sigma }\right){\left|\right|}_{F}$, where ||⋅||_*F*_ is the Frobenius norm. We have the following result:

**Theorem 1** (Davis-Kahan sin-*θ* theorem). *Let* $\Lambda ,\hat{\Lambda }\in R^{p\times p}$ *be symmetric matrices with eigenvalues* λ_1_ ≥ λ_2_ ≥ … ≥ λ_*p*_ *and* ${\hat{\lambda }}_{1}\geq {\hat{\lambda }}_{2}\geq \ldots \geq {\hat{\lambda }}_{p}$. *Let us fix* 1 ≤ *r* ≤ *s* ≤ *p and let d* = *s* − *r* + 1; *in addition, let* $\text{V}\in R^{p\times d}$ *and* $\hat{\text{V}}\in R^{p\times d}$ *be the two matrices spanned by the orthonormal eigenvectors* **e**_*r*_, **e**_*r*+1_, …, **e**_*s*_ *and* ${\tilde{\text{e}}}_{r},{\tilde{\text{e}}}_{r+1},\ldots ,{\tilde{\text{e}}}_{s}$ *of* **Λ** *and* $\tilde{\Lambda }$ *corresponding to eigenvalues* λ_*r*_, λ_*r*+1_, …λ_*s*_ *and* ${\tilde{\lambda }}_{r},{\tilde{\lambda }}_{r+1},\ldots {\tilde{\lambda }}_{s}$, *respectively*.

*Finally, set* $\delta =\text{inf}\{|\tilde{\lambda }-\lambda |:\lambda \in \left[\lambda_{s},\lambda_{r},\tilde{\lambda }\in \left(-\infty ,{\tilde{\lambda }}_{s+1}\right]\cup \left[{\tilde{\lambda }}_{s+1},+\infty \right)\right\}$ , *where we define* ${\tilde{\lambda }}_{0}=-\infty$, ${\tilde{\lambda }}_{p+1}=+\infty$ *and we assume δ* > 0. *Then*:

$$\text{sin}\left(\Theta \right)\left(\tilde{\text{V}},\text{V}\right)\leq \frac{\left|\right|\tilde{\Lambda }-\Lambda {\left|\right|}_{F}}{\delta }$$

It is possible to show that the equality above still holds true if we replace the Frobenius norm with the spectral norm [38]. We can set *r* = *s* = *j* = 1 to obtain:

$$\text{sin}\left(\Theta \right)\left({\tilde{\text{e}}}_{j},{\text{e}}_{j}\right)\leq \frac{\left|\right|\tilde{\Lambda }-\Lambda \left|\right|}{\text{min}\{|{\tilde{\lambda }}_{j-1}-\lambda_{j}|,|{\tilde{\lambda }}_{j+1}-\lambda_{j}|\}}$$

If we assume *j* = 1 (recall that λ_0_ = −∞), then

$$\text{sin}\left(\Theta \right)\left({\tilde{\text{e}}}_{1},{\text{e}}_{1}\right)\leq \frac{\left|\right|\tilde{\Lambda }-\Lambda \left|\right|}{|{\tilde{\lambda }}_{2}-\lambda_{1}|}$$

and, thus, we can get a bound on the norm of the difference.

Theorem 2 constructs a perturbation that turns the second largest eigenvalue of **A** into the largest one.

**Theorem 2**. *Let G be an undirected connected graph and let* **A** *be its adjacency matrix. Let* λ_1_ ≥ λ_2_ ≥ … ≥ λ_*n*_ *be the eigenvalues of* **A** *and let* **e**_1_, **e**_2_, …, **e**_*n*_ *be the corresponding eigenvectors. Let γ* = λ_1_ − λ_2_ *be the eigengap of* **A**. *For every ρ* > 0, *let us construct the perturbation* $\Delta \text{A}=[(1+\rho )\gamma ]{\text{e}}_{1}{\text{v}}_{1}^{T}$. *The largest eigenvalue and eigenvector of the matrix* $\tilde{\text{A}}=\text{A}-\Delta \text{A}$ *are* λ_2_ *and* **e**_2_, *respectively*.

*Proof*. Since *G* is an undirected graph, its adjacency matrix **A** is symmetric and its eigenvectors form an orthonormal basis in $R^{n}$. The eigendecomposition of **A** is as follows:

$$\text{A}=\sum_{l=1}^{n}\lambda_{l}{\text{e}}_{l}{\text{e}}_{l}^{T}$$

Let us choose *ρ* > 0 and let $\Delta \text{A}=[(1+\rho )\gamma ]{\text{e}}_{1}{\text{e}}_{1}^{T}$.

Because of the Cauchy-Schwartz inequality and since eigenvectors are orthonormal we have that $\|\Delta \text{A}{\|}_{F}=\|[(1+\rho )\gamma ]{\text{e}}_{1}{\text{e}}_{1}^{T}{\|}_{F}=(1+\rho )\gamma \|{\text{e}}_{1}{\text{e}}_{1}^{T}{\|}_{F}\leq (1+\rho )\gamma \|{\text{e}}_{1}\|\|{\text{e}}_{1}^{T}\|=(1+\rho )\gamma$.

The matrix $\tilde{\text{A}}=\text{A}-\Delta \text{A}$ admits the following expansion:

$$\begin{array}{c} \begin{array}{c} \tilde{\text{A}}=\sum_{l=1}^{n}\lambda_{l}{\text{e}}_{l}{\text{e}}_{l}^{T}-[(1+\rho )\gamma ]{\text{e}}_{1}{\text{e}}_{1}^{T}=\\ =\lambda_{1}{\text{e}}_{1}{\text{e}}_{1}^{T}-[(1+\rho )\gamma ]{\text{e}}_{1}{\text{e}}_{1}^{T}+\sum_{l=2}^{n}\lambda_{l}{\text{e}}_{l}^{T}{\text{e}}_{l}. \end{array} \end{array}$$

which can be further simplified as follows:

$$\begin{array}{c} \tilde{\text{A}}=[\lambda_{1}-(1+\rho )\gamma ]{\text{e}}_{1}{\text{e}}_{1}^{T}+\sum_{l=2}^{n}\lambda_{l}{\text{e}}_{l}^{T}{\text{e}}_{l} \end{array}$$

The last equality indicates that the eigenvalues of $\tilde{\text{A}}$ are λ_1_ − (1 + *ρ*)*γ*, λ_2_, λ_3_, …, λ_*n*_. By construction:

$$\begin{array}{c} \begin{array}{c} \lambda_{1}-(1+\rho )\gamma =\lambda_{1}-(1+\rho )(\lambda_{1}-\lambda_{2})=\\ =\lambda_{2}-\rho (\lambda_{1}-\lambda_{2})<\lambda_{2} \end{array} \end{array}$$

which implies that λ_2_ is the largest eigenvalue of $\tilde{\text{A}}$ and **e**_2_ is the associated eigenvector.

#### The *sensitivity* of Katz centrality

We conclude this piece of study with the Katz centrality. In particular, we will prove the following result:

**Theorem 3**. *Let G be an undirected and connected graph and let* **A** *be the adjacency matrix of G with eigenvalues* λ_1_ ≥ λ_2_ ≥ …λ_*n*_. *Suppose to construct a perturbed graph* $\tilde{G}$, *with adjacency matrix* $\tilde{\text{A}}=\text{A}-\Delta \text{A}$.

*The following results hold true*:

$$\frac{\left||\Delta \text{k}|\right|}{\left||\text{k}|\right|}\leq \frac{\beta ||\Delta \text{A}||}{1-\beta \lambda_{1}}$$

The previous theorem indicates us that the relative variation **k** *jointly depends on* ||**ΔA**||, the largest eigenvalue λ_1_ of **A** and the parameter *β*. Thus, if we assume that the product *βλ*_1_ approaches one, we may report a large variation in **Δk** even if we take ||**ΔA**|| < *ε*, for any positive and arbitrarily small constant *ε*.

We are now able to prove our result.

*Proof*. We will use the interpretation of the Katz coefficient **k** as the solution of a system of linear equations and, more specifically, our main tool to estimate **Δk** is the *conditioning number* [35], which can be defined as follows: consider a (vector) function *f*(*x*) and let us consider an infinitesimal variation *δx* of *x*; due to this variation, *f* changes too and let us call *δf*(*x*) the variation of *f*(*x*). The *relative condition number* *κ* = *κ*(*x*) is defined as

$$\kappa \left(x\right)=\underset{\delta (x)}{\text{sup}}(\frac{\frac{\left||\delta f(x)|\right|}{\left||f(x)|\right|}}{\frac{\left||\delta x|\right|}{\left||x|\right|}})$$

Assuming that *δx* is sufficiently small, the relative conditioning number identifies the largest value that the ratio of the variation of *f*(*x*) to the variation of *x* can take. Thus, the relative conditioning number acts as an upper bound on the variation of *f*(*x*) if we assume that *x* varies by at most *δx* (and *δx* is reasonably small).

In our case, the vector **k** is the solution of the linear system **Vk** = **1**. We recall that **V** = **I** − *β* **A** where $\text{A}\in R^{n\times n}$ is the adjacency matrix of *G*, $\text{I}\in R^{n\times n}$ is the *n* × *n* identity matrix and $\text{b}\in R^{n\times 1}$ is a *n*-th dimensional column vector whose entries are all equal one.

We assume to keep **b** fixed but to replace **V** with the matrix **V** + **ΔV** with **ΔV** sufficiently small. In this case, the solution **k** + **Δk** of the new system to solve is:

(V+ΔV)(k+Δk)=1→Vk+VΔk+ΔVk+ΔVΔk=1

By construction, we have that **Vk** = **1**; in the limit Δ*V* → 0 we can neglect the second order term **ΔVΔk** to obtain the following equality:

$$\text{V}\Delta \text{k}+\Delta \text{Vk}=0\to \text{V}\Delta \text{k}=-\Delta \text{Vk}\to \Delta \text{k}=-{\text{V}}^{-1}\Delta \text{Vk}$$

where the latter deduction is justified by the fact that the matrix **V** is non-singular for a suitable choice of *β*.

Because norms are sub-multiplicative, we conclude that:

$$\left||\Delta \text{k}|\right|=\left|\right|{\text{V}}^{-1}\Delta \text{Vk}||\to \left||\Delta \text{k}|\right|\leq \left|\right|{\text{V}}^{-1}\left||||\Delta \text{V}||||\text{k}|\right|$$

Theorem 12.2 in [35] enables us to rewrite the previous inequality in a more manageable fashion and, in particular, we have that the condition number *κ*(**V**) of the problem **Vk** = **1** is *κ*(**V**) = ||**V**||||**V**^−1^||.

If we combine the above result with the definition of the relative conditioning number, we obtain the following result:

$$\frac{\frac{\left||\Delta \text{k}|\right|}{\left||\text{k}|\right|}}{\frac{\left||\Delta \text{V}|\right|}{\left||\text{V}|\right|}}\leq \kappa \left(\text{V}\right)=\left||\text{V}|||\right|{\text{V}}^{-1}\left|\right|$$

which can be restated as follows:

$$\frac{\left||\Delta \text{k}|\right|}{\left||\text{k}|\right|}\leq \frac{\left||\Delta \text{V}|\right|}{\left||\text{V}|\right|}\kappa \left(\text{V}\right)$$

Since $\Delta \text{V}=(\text{I}-\beta \tilde{\text{A}})-(\text{I}-\beta \text{A})=(\text{I}-\beta (\text{A}-\Delta \text{A}))-(\text{I}-\beta \text{A})=\beta \Delta \text{A}$, we have that ||**ΔV**|| = *β*||**ΔA**||.

Because of **A** has eigenvalues λ_1_ ≥ λ_2_ ≥ … ≥ λ_*n*_, then the eigenvalues of **V** are 1 − *β*λ_1_, …, 1 − *β*λ_*n*_. The largest (resp., smallest) eigenvalue of **V** is 1 − *β*λ_*n*_ (resp., 1 − *β*λ_1_). Thus, we have that ||**V**|| = 1 − *β*λ_*n*_.

Analogously, **V**^−1^ has eigenvalues $\frac{1}{1-\beta \lambda_{1}},\ldots ,\frac{1}{1-\beta \lambda_{n}}$ and its largest (resp., smallest) eigenvalue is $\frac{1}{1-\beta \lambda_{n}}$ (resp., $\frac{1}{1-\beta \lambda_{1}}$).

The conditioning number of **V** is therefore:

$$\kappa \left(\text{V}\right)=\left||\text{V}|||\right|{\text{V}}^{-1}\left|\right|=\frac{1-\beta \lambda_{n}}{1-\beta \lambda_{1}}$$

We can combine the results above to get:

$$\frac{\Delta \text{k}}{\text{k}}\leq (\frac{\beta ||\Delta \text{A}||}{1-\beta \lambda_{n}})(\frac{1-\beta \lambda_{n}}{1-\beta \lambda_{1}})=\frac{\beta ||\Delta \text{A}||}{1-\beta \lambda_{1}}$$

which completes our proof.

### When a perturbation is small

**Fig 1:**
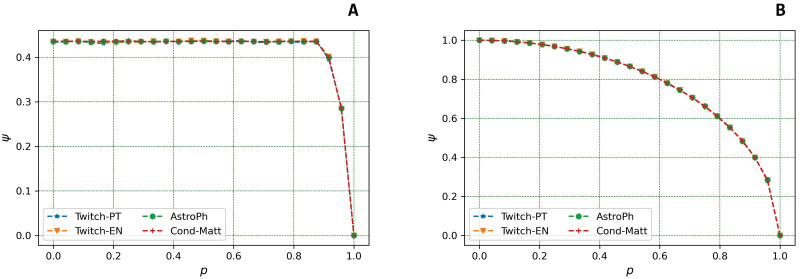
Variation of *ψ* in the *Uniform* model. The figure represents the variation of the evaluation metric *ψ* to quantify the amount of change in the adjacency matrix, as a function of the probability *p* for a node to fail, with *p* = [0, 1], in the *Uniform* model for the four real graphs under scrutiny if **(a)** the 10% of nodes are targeted to fail (*τ* = 0.1) or **(b)** if all the nodes are targeted (*τ* = 1).

**Fig 2:**
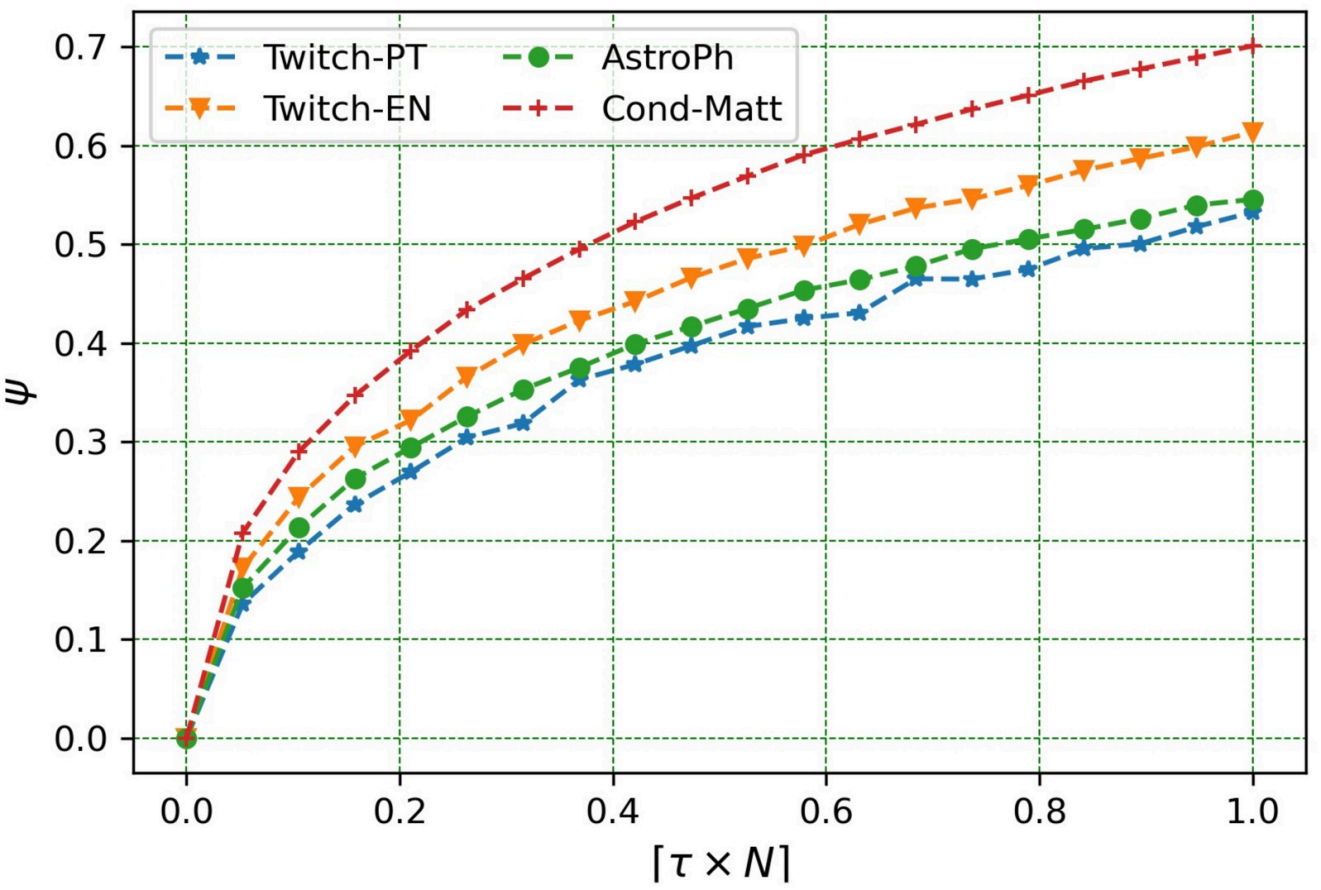
Variation of *ψ* in the *Best Connected* model. The figure represents the variation of the evaluation metric *ψ* to quantify the amount of change in the adjacency matrix, as a function of targeted nodes *τ* in the *Best Connected* model for the four real graphs under scrutiny.

In this section, the experiments related to answer to **RQ**_1_ are shown. Herein, indeed, we want to understand under which circumstances a perturbation on a graph *G* and, consequently, on its adjacency matrix **A** can be regarded as small [3]. Hence, we computed and evaluated the *ψ* variation (see Eq 3) as a function of *p* in the Uniform model (see Fig 1) and as a function of *τ* in the Best Connected Model (see Fig 2).

In our first analysis (see Fig 1) we focused on the network behaviour of the Uniform model. In Fig 1a and 1b we show our results for two extreme values of *τ*, namely: *τ* = 0.1 (*i.e*., we target only 10% of nodes) and *τ* = 1.0 (*i.e*., we target all nodes). In both plots, the dataset choice does not affect the *ψ* trend, which indicates that the parameter is independent from the dataset under exam. This is the reason why we have not used a wider range of datasets and this is in line with our experimental setup. Indeed, given a network with a fixed number of nodes and edges, it will be affected by a compensation effect because, after the perturbation, the outcome will be averaged among all the runs of the simulation and, thus, the topology considered for the experiment will stop to have a dominant role in the network perturbation (*i.e*., in the amount of change of the adjacency matrix).

The parameter *ψ* depends on the norm of **ΔA** that depends only on the product *τ*×*p* as well as on the graph order *n*; the dependence of ‖**ΔA**‖_*F*_ on *n* of nodes is absorbed by the denominator of *ψ*.

As we already discussed in [3], when we target a small fraction of nodes (*i.e*., *τ* = 0.1), then the *ψ* trend keeps constant up to *p* = 0.85; then, for *p* > 0.85, a steep decrease is noticed. This means that a small fraction of effectively failed nodes does not cause a relevant variation on the norm of the perturbation matrix.

This behaviour confirms and extends what already emerged in the state-of-art in the work by Albert *et al*. [40] on the robustness of random graphs upon random node removal, in which the authors proved that, if a small fraction of nodes is removed from an ER graph, a little variation of some topological graph’s properties emerged. Examples of such properties are the size of the largest connected component (*i.e*., the largest connected subgraph) or the graph’s diameter (*i.e*., the maximum length of the longest graph geodesic). The high level of resilience of ER graphs is still true for ‖**ΔA**‖_*F*_ [3, 40].

When all the nodes are targeted to potentially fail (*i.e*., *τ* = 1), the failure probability *p* (*i.e*., the higher *p*, the higher the likelihood the nodes actually fail) became crucial in perturbing *ψ*; indeed, we highlighted a decrease in *p* which gets more and more clear as *p* gets large [3].

In our second analysis we focused on the network behaviour of the Best Connected model shown by Fig 2, which displays the variation of *ψ* and as a function of *τ*. As expected, the higher *τ* (*i.e*., if *τ* → 1), the higher the likelihood of selecting high-degree nodes. Hence, picking and deleting high-degree nodes obviously causes a bigger increase in ‖**ΔA**‖_*F*_, as we already pinpointed in [3]. In the Best Connected model, indeed, the input graph topology significantly affects *ψ*. Then, the *ψ* trend increases almost linearly when *τ* grows, but the rate slightly differs from one dataset to another.

### How the centrality metrics vary in the two probabilistic failure models

The second experiment is proposed to address the **RQ**_2_. Hence, we wish to evaluate the variation of the centrality vector’s norm in the two probabilistic failure models under scrutiny.

In this section, we study how *ζ* varies as a function of *τ*. In the following simulations, *τ* is ranged from 0 to 0.2.

**Fig 3:**
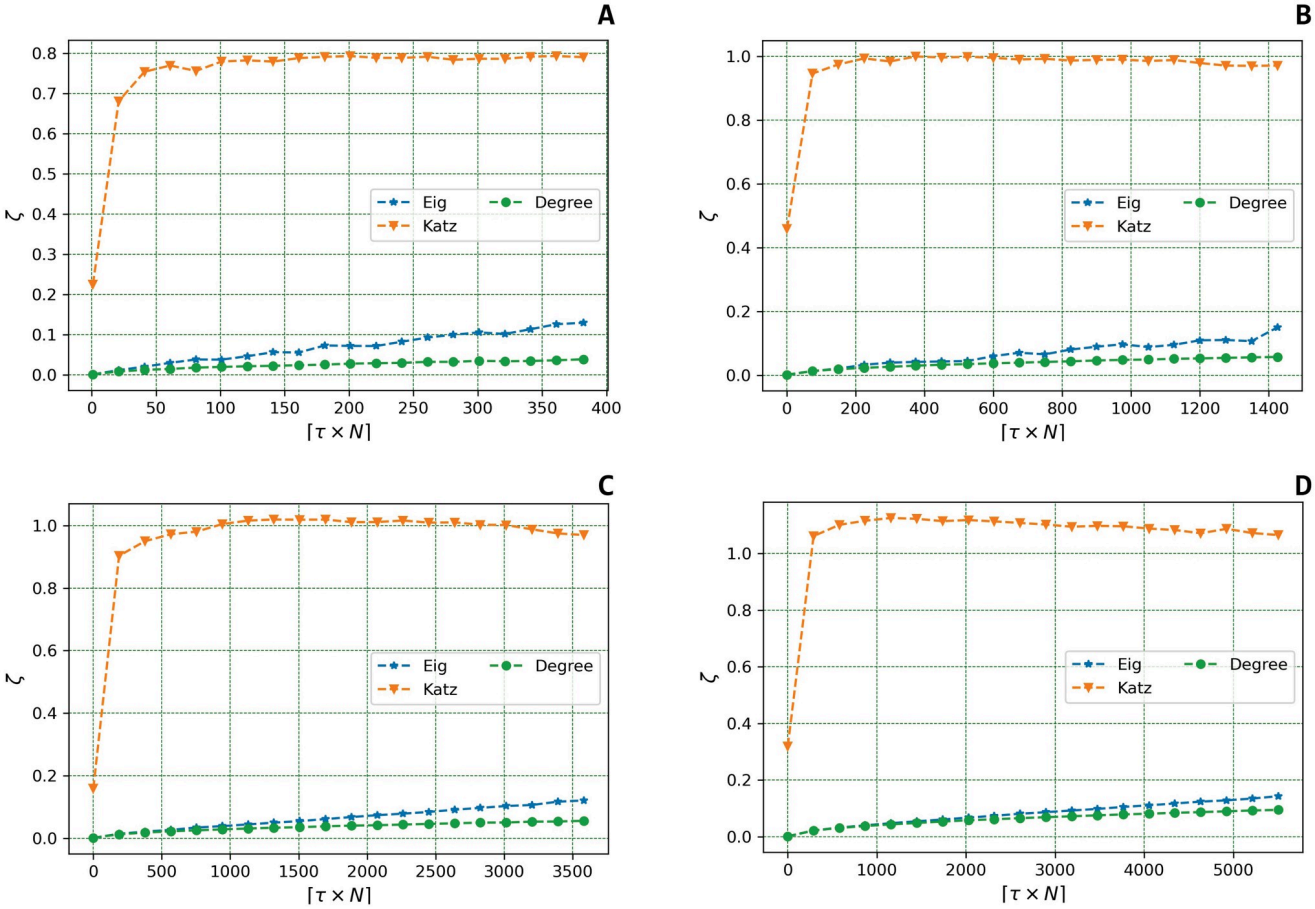
Variation of *ζ* in the *Uniform* model. The figures represent the variation of the evaluation metric *ζ*, which computes the deformation effect on the centrality metric under scrutiny, as a function of the fraction of targeted nodes *τ* in the *Uniform* model for the four real Datasets under scrutiny: **(a)** *Twitch PT*, **(b)** *Twitch EN*, **(c)** *AstroPh*, and **(d)** *Cond-Matt*.

**Fig 4:**
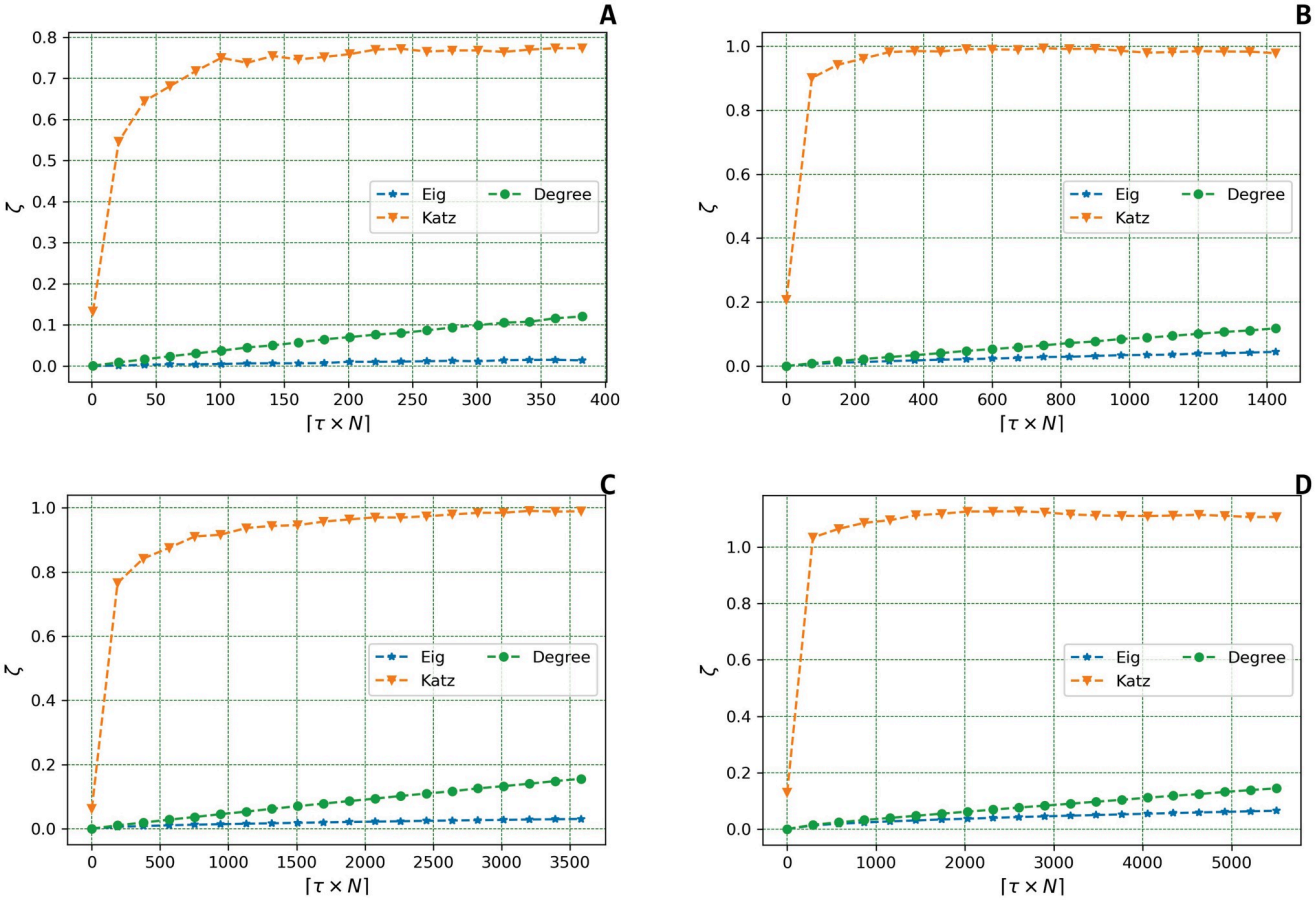
Variation of *ζ* in the *Best Connected* model. The figures represent the variation of the evaluation metric *ζ*, which computes the deformation effect on the centrality metric under scrutiny, as a function of the fraction of targeted nodes *τ* in the *Best Connected* model for the four real Datasets under scrutiny: **(a)** *Twitch PT*, **(b)** *Twitch EN*, **(c)** *AstroPh*, and **(d)** *Cond-Matt*.

Figs 3 and 4 show the variation of *ζ* in the Uniform and the Best Connected models, respectively. Note that, in the Uniform model, with reported herein the fixed failure probability of *p* = 0.1. We also defined *β* = 0.5 in the calculation of the Katz coefficient.

Despite the two different probabilistic models used to perform our analyses, we can highlight a similar trend. Indeed, we observe that both the Degree and the Eigenvector centrality increase in a linear fashion as *τ* increase contrary to the trend emerged with the Katz Centrality; herein, in fact, small values of *τ* are sufficient to generate a sharp increase in *ζ*. However, the observed values of *ζ* tend to quickly stabilise.

Furthermore, in the Uniform model, the *ζ* associated with the Eigenvector Centrality grows faster than the *ζ* corresponding to the Degree; an opposite trend emerges in the Best Connected model: herein, *ζ* grows faster in case of the Degree than in the Eigenvector Centrality [3].

### Computational analysis

We conclude our analysis by investigating the computational complexity of our approach to simulating node failure in a graph.

We first note that our approach can be divided into three distinct phases, namely: *a)* generating a perturbed graph *G*′ from an input graph *G*, *b)* computing the centrality measures (that is, the Degree, the Eigenvector and the Katz centrality) in *G*′, and *c)* comparing the centrality of each node in *G*′ and *G*.

As for phase *a)*, assume that *G* has *n* nodes and *m* edges: if *m* is of the same order of magnitude as *n*, then *G* is sparse; conversely, if *m* ≃ *n*^2^, then *G* is dense.

Let us then set the threshold *τ* and note that if we would apply either of the two methods described in the paper (Uniform and Best Connected) then we would produce a new graph *G*′ in which the number of nodes (and edges) is a function of both *τ* and the method we employed used to simulate node failure. Let *n*′ and *m*′ be the number of nodes and edges in *G*′.

To generate the nodes in *G*′, we select, uniformly at random, a subset of size ⌈*τn*⌉ from the nodes of *G*, and such an operation takes *O*(*n*), that is it is linear in the number of nodes of *G*; we then apply the Uniform/Best connected method on the candidate set above, and such an operation takes *O*(⌈*τn*⌉). It follows that the time needed to construct the set of nodes of *G*′ is proportional to *O*(⌈*τn*⌉ + *n*), which equals *O*(*n*) regardless of the failure method used.

As for the number *m*′ of edges in *G*′, we calculate the ratio *z* of *m*′ to *m*; the parameter *z* measures how much the size of *G*′ has been reduced with respect to *G* and it ranges between 0 (if no nodes and edges of *G* have been removed) and 1 (if all nodes and edges of *G* have been removed, thus generating an empty graph).

It follows that if *z* tends to 1, then a particular failure method (for a given *G* and a fixed value of *τ*) will generate a graph of small size, and, therefore, the subsequent steps *b)* and *c)* will necessarily be faster. The computation of *m*′ (and, thus of *z*) is much more difficult than the computation of *n*′ and it jointly depends on the topology of *G* and *τ*. The estimation of *m*′ cannot be approached analytically (except in some special cases) and we have therefore relied on appropriate simulations.

To this end, we have experimentally analysed the value of *z* for two special classes of graphs, namely random graphs generated according to the Erdős-Rényi (in short ER) model and random graphs generated according to the Barabási-Albert (in short, BA) model. In both cases, we have considered graphs of fixed size and, more specifically, each graph contained *n* = 10^6^ nodes.

As for ER graphs, we considered sparse graphs (here the probability *η* that two randomly chosen nodes are connected by an edge is equal to 10^−6^) and dense graphs (herein, the probability *η* that two randomly chosen nodes are connected by an edge is equal to 10^−4^).

Similarly, we have considered two configurations for graphs generated according to the BA model: in the first configuration we assume that a node can be connected to at most *q* = 2 nodes, while in the second, we assume that a node can be connected to at most *q* = 3 nodes.

**Fig 5:**
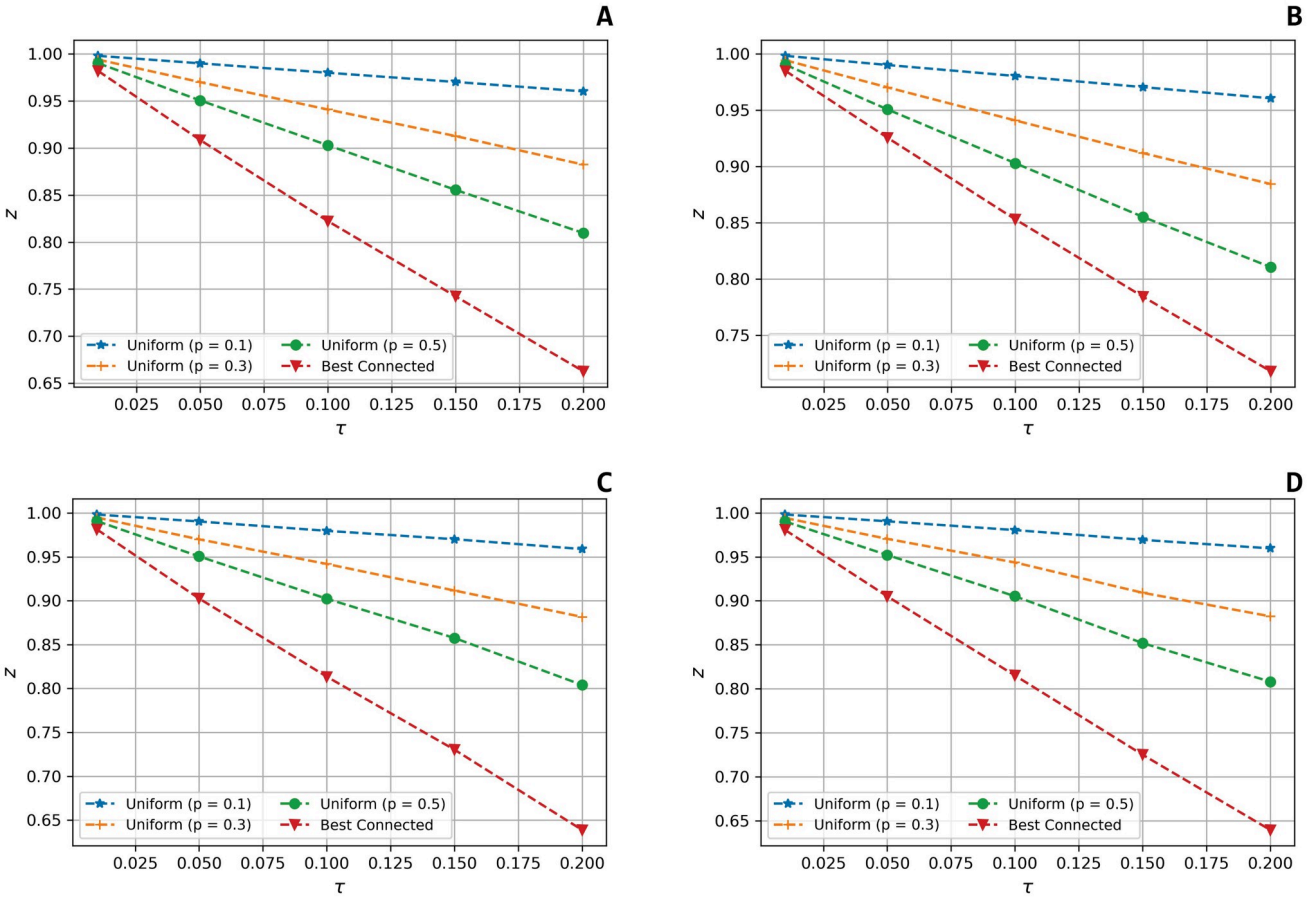
Variation of the parameter *z* as function of *τ* for ER and BA random graphs. The figure represents the variation of the parameter *z* as a function of *τ* performed in the *Uniform* (with failure probability equal to 0.1, 0.3, 0.5) and in the *Best Connected* models on: **(a)** an ER random graph with *n* = 10^6^ and *η* = 10^−6^, **(b)** an ER random graph with *n* = 10^6^ and *η* = 10^−4^, **(c)** a BA random graph with *n* = 10^6^ and *q* = 2, **(d)** and a BA random graph with *n* = 10^6^ and *q* = 3.

In Fig 5(a)–5(d) we graphically report how *z* varies when *τ* varies between 0.01 and 0.2 in the Uniform model (we assumed the failure probability of node is 0.1, 0.3 and 0.5, respectively) and in the Best Connected model.

Indeed, as for the tuning of *τ*, we want to avoid too-small values (otherwise the set of nodes to attack would be almost empty) and too-large values (otherwise the perturbation could no longer be classified as small). Hence, we have found that a value of *τ* ranging between 0.01 and 0.2 is reasonable to simulate node failures in real scenarios.

We note that in all the cases studied, *z* decreases linearly as *τ* increases, indicating that both the ER model (for all the values of the node failure probability we considered) and the Best Connected model are computationally realistic options to model node failures in large graphs.

As for step *b)*, we note that the computation of the degree is linear in the number of edges and, therefore, it takes *O*(*m*′) time. The eigenvector can be computed by using power iteration method [41] in *O*(*n* + *m*) time while the traditional methods to compute the Katz centrality takes *O*(*n*^3^) [42].

### Take-home message

There are plenty of studies in the state-of-art about the network error on the centrality vector and those types of problems are usually addressed by using rank correlation. However, the study herein presented goes beyond such purpose. Herein, we are not interested in understanding whether the ranking trend is the same across different centrality metrics, but we want instead to get insights on the *sensitivity* of the centrality metrics. Thus, our aim was to try to answer the following research question: *When a network is affected by a ‘small’ perturbation* (in our case, if a small portion of nodes up to 10% of the overall nodes is removed according to certain defined criteria)*, will the ranking of the centrality metrics be similarly perturbed or there will be huge variations in the rankings?*

Hence, we are not comparing the centrality metrics among each other, but we are comparing the network perturbation with its effects on each type of centrality metric herein considered. For the sake of simplicity, we considered, as a perturbation strategy, the removal of the nodes according to two probabilistic failure models (*i.e*., the *Uniform* and the *Best Connected*) [8]. However, different other strategies could be adopted otherwise with the same purpose of this study; to name a few, we could have removed edges, substructures, or communities. We wanted, instead, to quantify, if occurs, the dilatation effect in the centrality metrics ranking after a certain type of network perturbation.

To do so, after selecting the two probabilistic failure models to simulate the nodes’ failure (*i.e*., their removal from the original graph jointly with their incident edges) we investigated whether and to what extent small perturbations in a graph will affect the centrality metrics. Next, we selected two evaluation metrics to conduct our analyses, namely *ψ* (which quantifies the amount of change in the adjacency matrix due to the application of a perturbation) and *ζ* (which evaluate the deformation of centrality metrics), respectively.

From our tests conducted in the Uniform model emerged that, when a small fraction of nodes are targeted (*i.e*., *τ* = 10%), the perturbation is also small even up to a relatively high probability for the nodes to fail (*i.e*., failure probability *p* ≤ 85%). Moreover, we unveiled that the Eigenvector centrality is the most susceptible metric to deformation with respect to the other herein analysed [3].

From the analyses performed by using the Best Connected model, in contrast, we experienced that a higher fraction of targeted nodes can let to pick and successfully remove those strong nodes; thus, it results in a higher perturbation in the centrality metrics. A final consideration concerns the sensitivity of the centrality metrics; indeed, we noticed that, in this case, the most affected centrality metric is the Degree as the nodes’ resilience was established to be proportional to such a metric.

## Conclusions and future works

In this paper, we studied the *sensitivity* of some of the most used in literature centrality measures (*i.e*., Degree, Eigenvector and Katz Centrality) if some nodes in a graph fail to determine whether, after a ‘small’ perturbation of the network (*i.e*., a fixed fraction *τ* of nodes, where 0 < *τ* < 10% of the total number of nodes), the nodes will be kept almost in the same ranking positions. Indeed, re-ranking nodes after graph perturbations could be computationally onerous (*e.g*., it is an NP-hard problem) and, thus, it is a topic on which is worth gaining insights.

To perform our simulations, we used a probabilistic model in which a ‘small’ fraction of the top-ranked nodes in a graph may fail and the probability that a node fails follows a certain distribution. We considered two possible node failure distributions: *Uniform* (*i.e*., the probability of failure of each node is constant) and *Best Connected* (*i.e*., the node failure probability is proportional to node degree) [8].

From our analyses, which have been conducted on four real-world networks (*i.e*., Twitch-EN, Twitch-PT, AstroPh, and Cond-Mat), emerged that if *τ* is small, then the number of nodes that can actually fail is a small fraction of the entire node set and, thus, the failure of these nodes does not significantly affect the norm of the perturbation matrix. If *τ* = 1 all nodes can potentially fail; thus, the failure probability *p* (we recall that the highest *p*, the more likely the target node will be removed) became crucial to the variation of *ψ* (*i.e*., the evaluation metrics to quantify the network perturbation); specifically, we do not longer observe a range of values of *p* for which *ψ* remains constant, but we highlight a decrease in *p* which gets more and more clear as *p* gets large. As also highlighted in our previous work [3], in the Best Connected model simulations, contrary to what we experienced in the Uniform model, we noticed that the higher *τ* (*i.e*., if *τ* → 1), the higher the likelihood of selecting high-degree nodes. Hence, picking and deleting high-degree nodes obviously causes a bigger increase in ‖**ΔA**‖_*F*_. Indeed, the input graph topology significantly affects *ψ*. Then, the *ψ* trend increases almost linearly when *τ* grows, but the rate slightly differs from one dataset to another.

Our studies on the deformation of centrality metrics (*i.e*., *ζ*) unveiled that the Degree centrality is a *continuous* function, contrary to the Eigenvector one. Finally, the Katz centrality depends on how its *attenuation factor* *β* is tuned: for *β* < 1, Katz centrality behaves similarly to the Degree centrality; instead, for 1 < *β* < λ_1_, where λ_1_ is the *spectral radius*, it starts to behave more like the Eigenvector centrality and, thus, it is not a *continuous* function anymore.

Our next research goal consists of expanding the range of the pool of centrality metrics to study. Specifically, given their importance as tools for the analysis of complex systems, we plan to include centrality metrics such as the betweenness and the closeness. Unfortunately, the computation of betweenness/closeness relies on the calculation of all pairs shortest paths in a graph and, consequently, betweenness and closeness are hard to compute on graphs of even modest size. A nice option to consider is due to Behramand *et al*. [43], who introduce a centrality metric based on a node’s degree, its clustering coefficient, and the clustering coefficient of its second-level neighbours (*i.e*., the nodes that are two hops away from the node under study). The main observation of Behramand *et al*. [43] is that hubs/bridge nodes in a graph are likely to coincide with nodes with high degree and low clustering coefficient; moreover, if the sum of the second-level neighbours’ clustering coefficients of that node is large enough, then the second-level neighbours are located in a dense part of the graph. Putting all this information together, Behramand *et al*. [43] conclude that a node with low clustering, high degree, and dense second-level neighbors acts as a *structural hole* [44]; *i.e*. it connects different communities and thus has a privileged role in controlling the information spread over a graph. The proposed centrality metric can be efficiently implemented and scales linearly with the number of edges; moreover, it is effective in determining the nodes with the greatest spreading power, as experiments on real data suggest.

Some very interesting considerations on the choice of the centrality measure are reported in [5]. In this paper, Behramand *et al*. focus on *rich clubs*, *i.e*., subgroups of important or influential nodes that have been detected in a number of complex systems (*e.g*. transportation networks, scientific collaboration networks, and the human brain). Rich clubs play the role of the backbone of the network, optimising the routing of information to peripheral nodes. A nice result by Behramand *et al*. [5] is that if a graph contains a high rich-club, then degree centrality is the best tool to find influential nodes; however, such a rule does not apply to datasets with a low rich-club.

We note that the strategies discussed in this paper operate on nodes, but it would be possible to consider neutralisation strategies (especially removal strategies) that operate on edges [45]. For example, in the context of COVID-19 containment, measures to contain the virus spread before vaccines were available consist, for example, to close schools and public places (*e.g*., bars and restaurants) were largely implemented worldwide. Lockdown measures are aimed at eliminating social contacts and, therefore, they can be interpreted as edge deletion activities in the social network that encodes social contacts inside a community.

An interesting alternative to edge deletion is edge rewiring [46–49], which can be defined as: we remove two edges 〈*i*, *j*〉 and 〈*p*, *r*〉 from *G* and we add two new edges 〈*i*, *p*〉 and 〈*j*, *r*〉. Observe that in some cases the rewiring operation may change the degree distribution of the nodes; in other cases, we pretend that node degrees are preserved after edge rewiring. Some authors have studied extensively the problem of identifying an optimal edge rewiring, *i.e*., finding a sequence of rewiring operations to be performed on *G* in order to maximise its robustness [50–52] (which can be measured by various parameters such as algebraic connectivity, Randic coefficient or the number of spanning trees present in a graph). However, little is known about how rewiring might affect the centrality of nodes in a network, and whether rewiring itself has a stronger effect on some measures of centrality than others. We plan to fill this gap and, more precisely, we wish to study how the node centralities vary if edge rewiring is applied.

Another interesting research avenue requires to consider network simplification operations such as node aggregation: in this case, one or more nodes *u*_1_, … *u*_*k*_ are merged into a single supernode *u*. The analysis of node aggregation procedures is non-trivial since we are in charge of defining strategies that aggregate the failure probabilities of single nodes to obtain the failure probability of the corresponding supernode.
